# The Circ_0001367/miR-545-3p/LUZP1 Axis Regulates Cell Proliferation, Migration and Invasion in Glioma Cells

**DOI:** 10.3389/fonc.2021.781471

**Published:** 2021-11-18

**Authors:** Xuchen Dong, Peng Zhang, Liang Liu, Haoran Li, Shan Cheng, Suwen Li, Yuan Wang, Chaonan Zheng, Jun Dong, Li Zhang

**Affiliations:** ^1^ Department of Neurosurgery, Second Affiliated Hospital of Soochow University, Suzhou, China; ^2^ Medical College of Soochow University, Suzhou, China; ^3^ Department of Neurosurgery, Rugao Hospital Affiliated to Nantong University, Nantong, China; ^4^ College of Pharmaceutical Sciences, Soochow University, Suzhou, China

**Keywords:** circRNA, hsa_circ_0001367, miR-545-3p, LUZP1, glioma

## Abstract

Glioma is the most common primary intracranial malignant tumour in adults. It has a high incidence and poses a serious threat to human health. Circular RNA is a hotspot of cancer research. In this study, we aimed to explore the role of circ_0001367 in gliomagenesis and the underlying mechanism. First, qRT-PCR was conducted, which showed that circ_0001367 level was downregulated in glioma tissues and cells. Next, gain-of-function and loss-of-function assays were performed, which indicated that circ_0001367 inhibited the proliferation, migration and invasion of glioma cells. Subsequent bioinformatics analysis, dual-luciferase reporter assays, RNA immunoprecipitation assays and cell function assays demonstrated that circ_0001367 inhibited the proliferation, migration and invasion of glioma cells by absorbing miR-545-3p and thereby regulating the expression of leucine zipper protein (LUZP1). Finally, an *in vivo* experiment was conducted, which demonstrated that circ_0001367 inhibited glioma growth *in vivo* by modulating miR-545-3p and LUZP1. Taken together, the results of this study demonstrate that the circ_0001367/miR-545-3p/LUZP1 axis may be a novel target for glioma therapy.

## Introduction

Glioma, which originates from the malignant transformation of glial cells, is one of the most common primary intracranial malignant tumors in adults (1, 2). It has a high incidence and poses a serious threat to human health. According to the pathological characteristics of gliomas, the World Health Organization (WHO) classifies gliomas into four grades (I, II, III and IV) (3). Almost all glioma patients diagnosed with high-grade glioma (HGG, comprising grades III and IV) experience recurrence after surgical resection, and the median survival time of HGG patients is only 30~39 weeks (4). Although advances have been made in surgical resection, radiotherapy, chemotherapy and targeted therapy in recent decades, the therapeutic effects of glioma treatment, especially glioblastoma treatment, remain unsatisfactory (5). To improve the efficacy of glioma treatment, a greater understanding of the molecular mechanisms of gliomagenesis is urgently needed.

Circular RNAs (circRNAs) are a kind of non-coding RNA that are widespread in mammals and exhibit a circular structure formed by covalent bonds (6, 7). Researchers formerly considered circRNAs to be junk RNAs that do not play roles in regulating biological activities (8, 9). However, support for the competing endogenous RNA (ceRNA) hypothesis proposed by Pandolfi et al. overturned this conventional view, and an increasing number of biological functions of circRNAs are gradually being explored (10). The circRNAs involved in human disease progression mainly exert their regulatory roles through the following six mechanisms: 1) by acting as sponges or traps of microRNA (miRNA); 2) by acting as sponges or traps of protein; 3) by affecting the transcription of downstream genes; 4) by affecting the variable splicing of target genes; 5) by regulating the translation function of target proteins; and 6) by participating in the epigenetic modification of target genes or proteins (11, 12). Numerous studies have reported that circRNAs play important roles in glioma. Liu et al. showed that circHIPK3 promotes glioma progression by targeting miR-124 (13), and Lou et al. reported that CDR1as inhibits gliomagenesis by disrupting the p53/MDM2 complex (14). Furthermore, Yang et al. reported that circ-FBXW7 encodes a functional protein to regulate glioma progression (15). However, the potential role of hsa_circ_0001367, a novel circRNA located on chromosome 3 (q27.1), in glioma has not been fully studied.

MicroRNAs (miRNAs) are a class of endogenous non-coding RNAs that regulate gene expression by mediating post-transcriptional silencing (16, 17). MiRNAs are widely involved in cell proliferation, cell differentiation, immune responses and other processes, and increasing evidence shows that miRNAs are important regulatory factors in disease progression (18, 19). Recent studies suggest that miRNAs can serve as targets of circRNAs and thereby participate in regulating cancer progression (20). An analysis *via* an online database (starBase, http://starbase.sysu.edu.cn/index.php) indicated that miR-545-3p may be a target of hsa_circ_0001367; however, the regulatory network containing miR-545-3p and hsa_circ_0001367 has not been reported.

Leucine zipper protein (LUZP1), predominantly expressed in brain, is reportedly involved in many diseases (21–23); however, its role in glioma remains unknown. Data from starBase indicate that the 3’-untranslated regions (3’-UTRs) of LUZP1 contain binding sites of miR-545-3p. In this study, we detected and correlated the expression of hsa_circ_0001367, miR-545-3p and LUZP1 in glioma tissues and cell lines. Moreover, function assays were performed to explore the mechanisms underlying the roles of hsa_circ-0001367/miR-545-3p/LUZP1 in gliomagenesis.

## Materials and Methods

### Patient Enrollment

Glioma tissues and samples of corresponding adjacent normal brain tissue (36 paired samples) were obtained from patients diagnosed with glioma and admitted to Soochow University. The tissues were stored in liquid nitrogen immediately after excision. Written informed consent was obtained from all the glioma patients who participated in this study.

### Cell Culture and Transfection

Human normal astrocytes were purchased from Jennio Biological Technology (Guangzhou, China). Human glioma cell lines U87, LN229, U251 and T98G were purchased from Procell (Wuhan, China). All the cell lines were cultured in Dulbecco’s modified Eagle’s medium containing 10% fetal bovine serum (FBS, Gibco, USA). The cell lines were maintained in a humidified incubator (37°C and 5% CO_2_). Small interfering RNA (siRNA), miR-545-3p mimic, miR-545-3p inhibitor, overexpression plasmid (full-length sequence of circ_0001367 was cloned into pGL3-Basic Vector) and the corresponding negative controls used in this study were all synthesized by GenePharma (Shanghai, China). Once the cells reached approximately 80% confluence, they were transfected with synthesized oligonucleotides using Lipofectamine 3000 (Invitrogen, USA) according to the manufacturer’s protocol.

### Quantitative Real-Time Polymerase Chain Reaction (qRT-PCR)

Total RNA was isolated from tissues and cells by using TRIzol (Invitrogen, USA). RNA was reverse transcribed into complementary DNA with the First Strand cDNA Synthesis Kit (MBI, Canada). SYBR Green Master Mix II (Takara, Japan) and the ABI 7900 system (Applied Biosystems, USA) were used to conduct qRT-PCR according to the manufacturers’ protocols. GAPDH and U6 were used as controls. The expression level of targets was determined by 2^−ΔΔCt^ method. The following primers used in this study. Circ_0001367, 5′-TGG GTC TAT CGT GCC GTT GA-3′ (forward) and 5′-GGA CAT CAT TTC ATT CCC AAG TA-3′ (reverse); miR-545-3p, 5′-TGG CTC AGT TCA GCA GGA AC-3′ (forward) and 5′-TGG TGT CGT GGA GTCG-3′ (reverse); LUZP1, 5′-ATG GCC GAA TTT ACA AGC TAC-3′ (forward) and 5′-TCA GTT CTC CTC AGC ACA GG-3′ (reverse); GAPDH, 5′-CAT CAC TGC CAC CC AG-3′ (forward) and 5′-ATG CCAG TGA GCT TC CC-3′ (reverse); U6, 5′-CTC GCT TCG GCA GCA CA-3′ (forward) and 5′-AAC GCT TCA CGA ATT TGC GT-3′ (reverse).

### Western Blot

Total protein was extracted from cells with the Total Protein Extraction Kit (KeyGEN BioTECH, Shanghai, China). Protein concentration was determined with the BCA Protein Assay Kit (KeyGEN BioTECH, Shanghai, China). Then, the proteins were separated by 10% SDS-PAGE and transferred to nitrocellulose membranes. After being blocked with blocking buffer for 10 min, the membranes were incubated with primary antibodies against LUZP1 (Proteintech, USA) and GAPDH (Proteintech, USA) overnight at 4°C. The membranes were then incubated with horseradish peroxidase-conjugated secondary antibody at room temperature for 1 h. Image Quant LAS (GE, USA) was used for signal detection.

### Dual-Luciferase Reporter Assay

The wild-type (WT) and mutant (MUT) sequences of circ_0001367 and miR-545-3p were synthesized and inserted into pmirGLO vectors. Next, LN229 and T98G cells were transfected with miR-545-3p mimic or NC mimic along with WT or MUT reporter plasmids by using Lipofectamine 3000 (Invitrogen, USA). After 48 h, the luciferase activity was determined by the Dual-Luciferase Reporter Assay (Promega, USA).

### Terminal Deoxynucleotidyl Transferase dUTP Nick End Labeling (TUNEL) Staining

TUNEL staining was conducted by using the TUNEL Assay Kit (Beyotime, Shanghai, China). Cells were fixed with anhydrous ethanol and washed three times with PBS. Next, the cells were successively treated with Triton-X-100, terminal deoxynucleotide transferase (TdT), streptavidin HRP solution and DAB substrate according to the manufacturer’s protocol. The results were evaluated *via* microscope (Nikon, Japan).

### RNase R Treatment

After being isolated from LN229 and T98G cells, total RNA was incubated with RNase R or the mock treatment for 30 min (37°C). Then, the expression of circ_0001367 and GAPDH was determined by qRT-PCR (24).

### Actinomycin D Assay

Actinomycin D (2 mg/ml, APExBIO, USA) was added to culture medium to culture LN229 and T98G cells for 24 h. Next, the cells were harvested, and their expression of circ_0001367 and GAPDH was determined by qRT-PCR (24).

### 3-(4,5-Dimethylthiazol-2-yl)-2,5-Diphenyltetrazolium Bromide (MTT) Assay

MTT assays were performed with an MTT kit (Beyotime, Shanghai, China). Cells were incubated with MTT solution (20 µl, 5 mg/ml) for 0, 24, 48 or 72 h. Then, dimethyl sulfoxide (DMSO, 4% 200 µl, Beyotime, Shanghai, China) was added to the cells. The absorbance at 490 nm was then determined with a microplate reader (Bio-Tek, Germany).

### 5-Ethynyl-20-Deoxyuridine (EdU) Assay

EdU assays were performed by using the EdU Cell Proliferation Kit with Alexa Fluor 488 (Beyotime, Shanghai, China). Cells were incubated with culture medium and EdU labelled for 4 h. After being fixed with anhydrous ethanol and treated with Triton-X-100, the cells were incubated with reaction buffer for 30 min and stained with DAPI for 5 min in darkness. The number of EdU-positive cells was measured with a fluorescence microscope (Olympus, Japan).

### Transwell Assay

Cells were suspended in FBS-free culture medium (200 μl) and added to the upper chamber of Transwell inserts (Millipore, Germany). Medium (500 μl) containing 10% FBS was added to the lower chamber. After incubation in a humidified incubator (37°C and 5% CO_2_) for 24 h, the non-migrated cells in the upper chamber were wiped with a wet cotton swab. The remaining cells were fixed in anhydrous ethanol and stained with 0.5% crystal violet. After being washed with PBS, the stained cells were imaged and counted under a microscope (Nikon, Japan). The invasion assay was conducted following the same steps described above for the migration assay except that the chambers were precoated with Matrigel (BD Biosciences, USA).

### Tumor Xenograft Experiment

Nude mice aged 4-5 weeks were obtained from Shanghai SLAC Laboratory Animal Company (Shanghai, China). Stable cell lines LN229 overexpressing circ_0001367 or corresponding negative control were administered into the dorsal surface of the mice shoulder. Three weeks later, the tumors formed in the mice were excised. Tumor volume and weight were determined (volume = 0.5 ×length×width^2^). In addition, the expression of circ_0001367, miR-545-3p and LUZP1 was determined by qRT-PCR, western blot or immunohistochemistry staining.

### Statistical Analysis

The results of this study are presented as the mean ± standard deviation. SPSS software 19.0 (SPSS, USA) was used for statistical analysis. Student’s t-test was used for comparisons between groups. P <0.05 was considered statistically significant.

## Results

### Hsa_circ_0001367 Was Downregulated in Glioma

**Figure 1:**
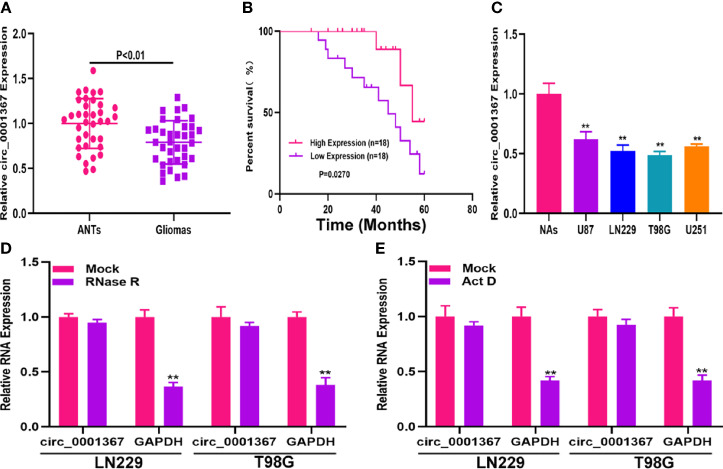
Hsa_circ_0001367 was downregulated in glioma. **(A)** Expression of hsa_circ_0001367 in glioma tissues and adjacent non-neoplastic tissues (ANTs) detected by qRT-PCR. **(B)** Survival was compared between patients with high and low levels of hsa_circ_0001367 by Kaplan-Meier analysis. **(C)** Expression of hsa_circ_0001367 in glioma cell lines and normal human astrocytes (NHAs). **(D)** Expression of hsa_circ_0001367 and GAPDH in T98G and LN229 cells treated with RNase R or the mock treatment. **(E)** Expression of hsa_circ_0001367 and GAPDH in T98G and LN229 treated with Actinomycin D (Act D) or Mock. **P < 0.01.

qRT-PCR was used to measure circ_0001367 in the clinical samples. As shown in **Figure 1A**, circ_0001367 was significantly downregulated in glioma tissues compared with adjacent normal brain tissues (ANTs). To determine whether circ_0001367 expression was associated with clinical features, Kaplan-Meier analysis was performed. The results indicated that patients in the low circ_0001367-expression group had a shorter survival time than those in the high-expression group (**Figure 1B**). In addition, we detected circ_0001367 expression in glioma cell lines and found that circ_0001367 was obviously downregulated in glioma cell lines compared with NAs (**Figure 1C**). RNase R digestion demonstrated that circ_0001367 was resistant to RNase R (**Figure 1D**). In addition, actinomycin D treatment showed that the circular structure of circ_0001367 was stable (**Figure 1E**). These findings suggested that circ_0001367 is involved in gliomagenesis.

### Hsa_circ_0001367 Inhibits the Proliferation, Migration and Invasion of Glioma Cells

**Figure 2:**
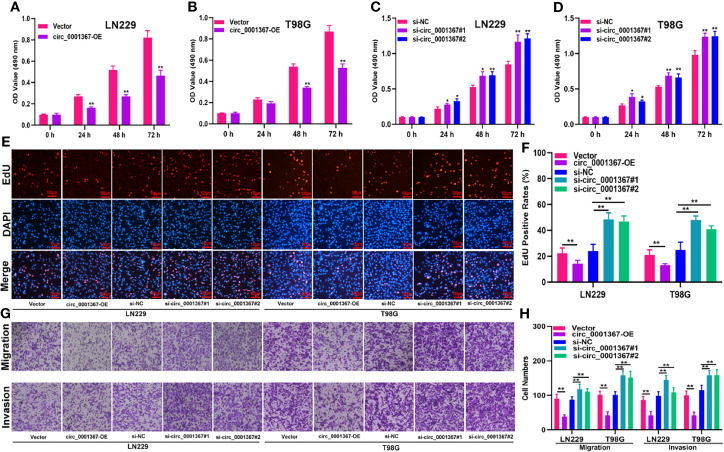
Hsa_circ_0001367 inhibits the proliferation, migration and invasion of glioma cells. **(A–D)** The effect of circ_0001367 on cell viability in T98G and LN229 cells was assessed with MTT assays. **(E, F)** EdU assays were used to evaluate the effect of circ_0001367 on the proliferation of T98G and LN229 cells. **(G, H)** Transwell assays were performed to analyse the effects of circ_0001367 on the migration and invasion of T98G and LN229 cells. *P < 0.05, **P < 0.01.

To explore the role of circ_0001367 in gliomagenesis, circ_0001367 was upregulated or knocked down in LN229 and T98G cells by transfecting the cells with overexpression plasmid or small interfering RNA (siRNA). The transfection efficiency was verified by qRT-PCR (**Figures S1A, B**). MTT assays and EdU assays demonstrated that circ_0001367 overexpression obviously suppressed the proliferation of glioma cells. In contrast, the proliferation capacity of glioma cells was markedly promoted in circ_0001367-knockdown groups (**Figures 2A–F**). Furthermore, Transwell assays were performed to assess the effects of circ_0001367 on the migration and invasion of glioma cells. We found that circ_0001367 overexpression significantly inhibited the migration and invasion ability of glioma cells, whereas circ_0001367 knockdown markedly enhanced the migration and invasion of glioma cells (**Figures 2G, H**). Taken together, these results suggest that circ_0001367 inhibits the proliferation, migration and invasion of glioma cells *in vitro*.

### Hsa_circ_0001367 Act as a Sponge of miR-545-3p

**Figure 3:**
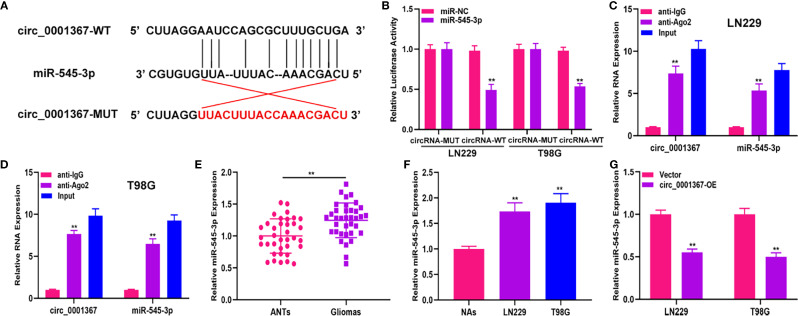
Hsa_circ_0001367 act as a sponge of miR-545-3p. **(A)** The binding sites between hsa_circ_0001367 and miR-545-3p. **(B)** Dual-luciferase reporter assay showed that miR-545-3p could more greatly decrease the luciferase activity of circ_0001367-WT than circ_0001367-MUT. **(C, D)** RIP assay was performed to verify the relationship between circ_0001367 and miR-545-3p. **(E)** qRT-PCR was used to detect the expression of miR-545-3p in clinical samples. **(F)** qRT-PCR was used to detect the expression of miR-545-3p in NHAs and glioma cell lines. **(G)** The expression of miR-545-3p in T98G and LN229 cells transfected with circ_0001367 plasmid was detected by qRT-PCR. **P < 0.01.

The data obtained from starBase showed the presence of binding sites between circ_0001367 and miR-545-3p (**Figure 3A**). The luciferase reporter assay indicated that miR-545-3p was able to obviously reduce the luciferase activity of circ_0001367-WT cells; however, it had no pronounced effect in the circ_0001367-MUT group (**Figure 3B**). Furthermore, RIP assay verified that circ_0001367 and miR-545-3p were enriched in the anti-Ago2 group (**Figures 3C, D**). Next, we detected the expression of miR-545-3p in glioma tissues and cell lines. The results showed that miR-545-3p was upregulated in glioma tissues and cell lines compared with ANTs and NAs, respectively (**Figures 3E, F**). We also detected the level of miR-545-3p in circ_0001367-overexpression LN229 and T98G cells. The results demonstrated that miR-545-3p was downregulated when circ_0001367 was overexpressed (**Figure 3G**). These findings suggest that circ_0001367 acts as a sponge of miR-545-3p.

### Hsa_circ_0001367 Suppresses Glioma Progression by Absorbing miR-545-3p

**Figure 4:**
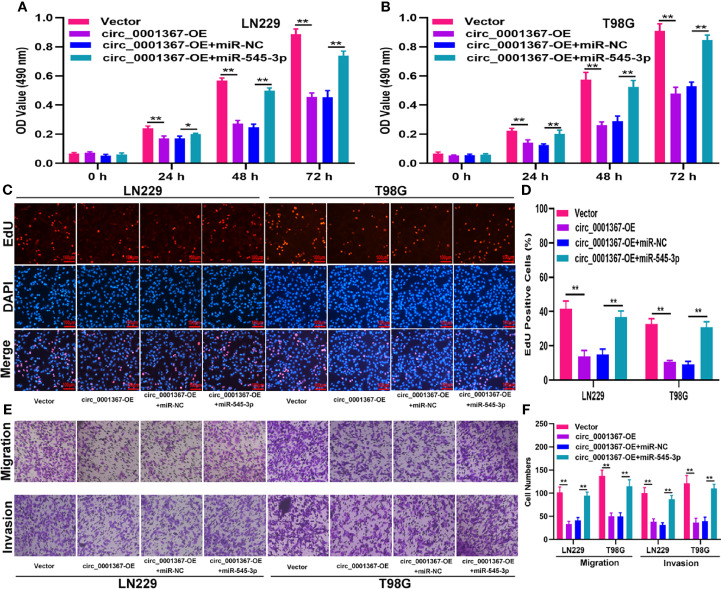
Hsa_circ_0001367 suppresses glioma progression by absorbing miR-545-3p. **(A, B)** Cell proliferation was investigated by MTT assay after transfection. **(C, D)** Cell proliferation was tested by EdU assay after transfection. **(E, F)** Transwell assay was conducted to measure the migration and invasion capacity of T98G and LN229 cells after transfection. *P < 0.05, **P < 0.01.

To investigate whether circ_0001367 exerts its function in LN229 and T98G cells by sponging miR-545-3p, function assays were conducted. The transfection efficiency of circ_0001367-overexpression plasmid and miR-545-3p mimics was verified by qRT-PCR (**Figure S1C**). The MTT assays and EdU assays demonstrated that circ_0001367 overexpression obviously decreased the proliferation of glioma cells and that this inhibitory effect could be reversed by miR-545-3p mimic (**Figures 4A–D**). Similarly, the Transwell assays showed that miR-545-3p mimic could reverse the inhibition of migration and invasion caused by circ_0001367 overexpression (**Figures 4E, F**). These results suggest that circ_0001367 suppresses the proliferation, migration and invasion of glioma cells by absorbing miR-545-3p.

### LUZP1 Is the Direct Target of miR-545-3p

**Figure 5:**
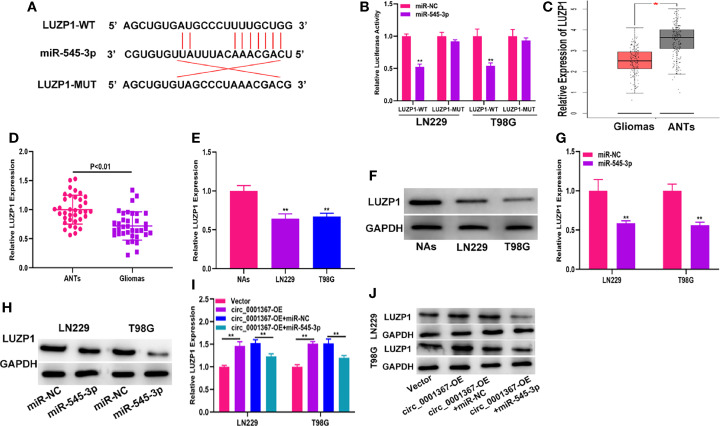
LUZP1 is the direct target of miR-545-3p. **(A)** The binding sites of miR-545-3p and LUZP1 predicted by starBase. **(B)** The relative luciferase activity in T98G and LN229 cells was detected by dual-luciferase reporter assay. **(C)** The expression of LUZP1 in the public database (GEPIA). **(D)** The expression of LUZP1 in clinical samples detected by qRT-PCR. **(E, F)** The expression of LUZP1 in glioma cell lines detected by qRT-PCR and western blot. **(G, H)** LUZP1 expression in T98G and LN229 which transfected with miR-NC or miR-545-3p detected by qRT-PCR and western blot. **(I, J)** LUZP1 expression in T98G and LN229 cells transfected with vector, circ_0001367, circ_0001367+miR-NC or circ_0001367+miR-545-3p was detected by qRT-PCR and western blot. *P < 0.05, **P < 0.01.

The data from starBase indicated that the 3’-UTR of LUZP1 contains sites that can bind to miR-545-3p (**Figure 5A**). The luciferase reporter assay indicated that miR-545-3p could reduce the luciferase activity of LUZP1-WT cells much more than that of LUZP1-MUT cells (**Figure 5B**). By searching the online database GEPIA, we found that LUZP1 was downregulated in glioma tissues (**Figure 5C**). Next, we detected LUZP1 expression in clinical samples by qRT-PCR. The results demonstrated that LUZP1 was downregulated in glioma tissues compared with ANTs (**Figure 5D**). Consistent with this finding, LUZP1 was downregulated in LN229 and T98G cells compared with NAs (**Figures 5E, F**). Furthermore, we detected LUZP1 expression in the cell models we constructed previously. qRT-PCR and western blot indicated that hsa_circ_0001367 overexpression could promote expression of LUZP1, meanwhile, miR-545-3p mimic could suppress expression of LUZP1 (**Figures 5G–J**). These findings indicate that LUZP1 is the direct target of miR-545-3p.

### LUZP1 Downregulation Restored the Effect of miR-545-3p Knockdown or circ_0001367 Overexpression on Glioma Cells

**Figure 6:**
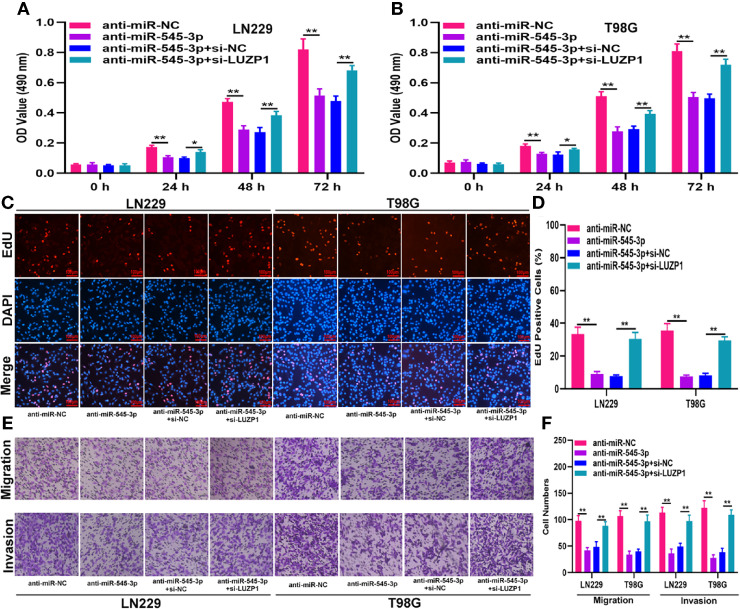
LUZP1 downregulation restored the effect of miR-545-3p knockdown on glioma cells. **(A, B)** The proliferation of indicated cells was tested by MTT. **(C, D)** The proliferation of indicated cells was tested by EdU. **(E, F)** Transwell assay was conducted to measure the migration and invasion capacity of T98G and LN229 cells after transfection. *P < 0.05, **P < 0.01.

**Figure 7:**
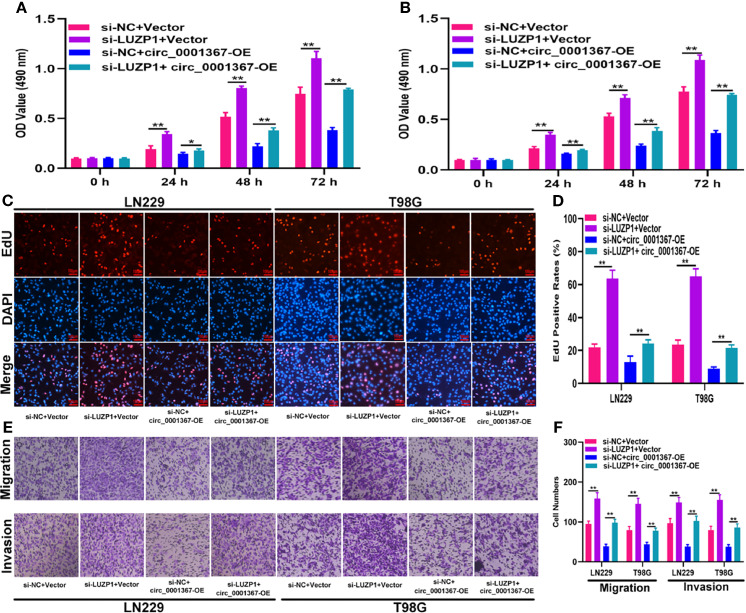
LUZP1 downregulation restored the effect of circ_0001367 overexpression on glioma cells. **(A, B)** The proliferation of cells transfected with si-LUZP1, circ_0001367 or si-LUZP1 plus circ_0001367 was tested by MTT assay. **(C, D)** The proliferation of cells transfected with si-LUZP1, circ_0001367 or si-LUZP1 plus circ_0001367 was tested by EdU assay. **(E, F)** Transwell assay was conducted to measure the migration and invasion capacity of cells which transfected with si-LUZP1, circ_0001367 or si-LUZP1 plus circ_0001367. *P < 0.05, **P < 0.01.

To investigate whether LUZP1 is the functional target of miR-545-3p in LN229 and T98G cells, we constructed four cell models. The efficiency of miR-545-3p inhibitor and si-LUZP1 transfection was verified by qRT-PCR (**Figure S1D**). The MTT and EdU assays suggested that LUZP1 deletion could block the suppressive effect of miR-545-3p inhibitor on glioma proliferation (**Figures 6A–D**). Consistent with this finding, the Transwell assays showed that the migration and invasion ability of LN229 and T98G cells was impaired by miR-545-3p inhibitor, whereas LUZP1 deletion reversed this inhibitory effect (**Figures 6E, F**). In addition, function assays indicated that LUZP1 deletion could block the suppressive effect of circ_0001367 overexpression on glioma proliferation, migration and invasion (**Figures 7A–F**). These results suggested that LUZP1 downregulation restored the effect of miR-545-3p knockdown and circ_0001367 overexpression on glioma cells.

### Hsa_circ_0001367 Inhibited Glioma Growth *In Vivo*

**Figure 8:**
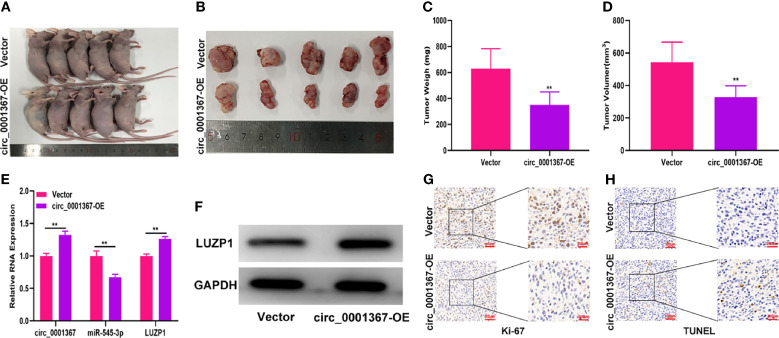
Hsa_circ_0001367 inhibited glioma growth *in vivo*. **(A, B)** Tumour formation was examined in nude mice following the implantation of LN229 xenografts with vector or circ_0001367 overexpression. **(C, D)** The volume and weight of tumours formed in the nude mice. **(E)** The expression of circ_0001367, miR-545-3p and LUZP1 in the tumours formed in the nude mice was measured by qRT-PCR. **(F)** The expression of LUZP1 in the tumours formed in the nude mice was measured by western blotting. **(G)** Immunohistochemistry for Ki-67 in the tumours formed in the nude mice. **(H)** TUNEL staining of the tumours formed in the nude mice. **P < 0.01.

To evaluate the effect of circ_0001367 on glioma growth *in vivo*, LN229 cells transfected with circ_0001367-overexpression plasmid or corresponding negative control were subcutaneously injected into mice. Three weeks later, tumors had formed. Compared with those in the negative control group, the tumors formed in the circ_0001367-overexpression group were lower in weight and smaller in volume (**Figures 8A–D**). qRT-PCR showed that circ_0001367 was upregulated whereas miR-545-3p was downregulated in the tumors formed in the circ_0001367-overexpression group (**Figure 8E**). Furthermore, qRT-PCR and western blot revealed that LUZP1 was upregulated in the circ_0001367-overexpression group relative to the control group (**Figures 8E, F**). Furthermore, Ki-67 staining indicated that circ_0001367 overexpression decreased the proportion of Ki-67-positive cells (**Figure 8G**). TUNEL staining showed that more apoptosis occurred in the circ_0001367-overexpression group than in the control group (**Figure 8H**).

## Discussion

Glioma is the most common malignant tumor of the central nervous system. In the past 30 years, despite numerous studies on glioma pathogenesis, the prognosis of glioma patients has not improved significantly (25). Therefore, it is of great significance for the clinical diagnosis and treatment of glioma to focus on the pathogenesis of glioma.

CircRNAs have become a hot topic in cancer research since the recent replacement of the traditional view of circRNAs as non-functional molecules with the understanding that they influence various cellular activities (9, 26). CircRNAs are not sensitive to nucleases, so it is more stable than the corresponding linear RNAs, which makes them a significant advantage as a new clinical diagnostic marker and target for therapy (27). Accumulating studies have shown that several circRNAs are closely associated with human cancers. For example, circ_0000039 has been found to be a biomarker of tissue differentiation in gastric cancer (28); circ_0000282 has been shown to be upregulated in osteosarcoma and associated with the proliferation of osteosarcoma cells (29); and circ_0026416 functions as an oncogene in colorectal cancer and may be a novel biomarker for diagnosis (30). In the present study, we found that circ_0001367 was significantly downregulated in glioma tissues and cells and that its expression level was negatively associated with the survival of glioma patients. The gain-of-function and loss-of-function assays revealed that circ_0001367 overexpression inhibits the proliferation, migration and invasion of glioma cells, whereas circ_0001367 silencing promotes these processes. These findings suggest that circ_0001367 acts as a suppressor in glioma.

It is currently theorized that circRNAs are mainly distributed in the cytoplasm and play a role in regulating tumor progression through the adsorption of miRNAs. For example, circ_0001785 can sponge miR-942 or miR-1200 to regulate breast cancer or osteosarcoma progression, respectively (31, 32); and circ_0000285 can absorb miR-409-3p, miR-197-3p and miR-599, thereby affecting the progression of cervical cancer and osteosarcoma (24, 33, 34). Analysis using an online database revealed 29 miRNAs that may serve as the target of circ_0001367. Among them, miR-545-3p aroused our great interest. MiR-545-3p dysregulation has been reported in diverse cancers. Shi et al. reported that miR-545-3p was downregulated in epithelial ovarian cancer (35); Hao et al. showed that miR-545-3p inhibits the proliferation and differentiation of osteoblasts (36); and Zhong et al. reported that miR-545-3p functions as a tumor suppressor in endometrial carcinoma (37). Furthermore, some studies indicate that miR-545-3p may be the target of circRNAs. Chen et al. reported that miR-545-3p can be sponged by circ_0007580 and is thus involved in non-small lung cancer progression (38); and Li et al. showed that circ_0072083 promotes non-small lung cancer progression by absorbing miR-545-3p (39). To date, the role of miR-545-3p in glioma and the associated circRNA-miRNA network have not been studied. In our study, employing a dual-luciferase reporter assay and RIP assay, we verified that miR-545-3p can be sponged by circ_0001367. In addition, function assays indicated that miR-545-3p can reverse the effects of circ_0001367 overexpression on glioma proliferation, migration and invasion. All of these findings suggest that circ_0001367 sponges miR-545-3p to regulate gliomagenesis.

Using an online database, we found that miR-545-3p directly targets LUZP1. LZUP1 is a regulator of primary cilia and actin cytoskeleton and has been reported to be associated with colorectal cancer, neural tube closure and Townes-Brocks syndrome (21–23); however, its role in glioma has not been studied. Herein, we showed that LUZP1 was significantly downregulated in glioma tissues and cells. Furthermore, function assays indicated that LUZP1 functions as a suppressor in glioma and is regulated by miR-545-3p.

In summary, our study revealed that circ_0001367 was markedly downregulated in glioma tissues and cells. In addition, it demonstrated that circ_0001367 suppresses the proliferation, migration and invasion of glioma cells by sponging miR-545-3p to regulate LUZP1. The circ_0001367/miR-545-3p/LUZP1 axis may be a novel target for glioma therapy.

## Data Availability Statement

The original contributions presented in the study are included in the article/**Supplementary Material**. Further inquiries can be directed to the corresponding authors.

## Ethics Statement

The animal study was reviewed and approved by Ethics Committee of Soochow University.

## Author Contributions

XD conducted the cell assays and wrote the manuscript. PZ, HL, and CZ collected clinical samples and conducted the *in vivo* experiments. LL, SC, and SL analyzed the data, search literatures and prepared the figures. YW designed the experiments and edited the manuscript. LZ designed the experiments, supervised the research and reviewed the manuscript. All authors contributed to the article and approved the submitted version.

## Funding

This study was supported by Hui-Chun Chin and Tsung-Dao Lee Chinese Undergraduate Research Endowment (CURE); National Natural Sciences Foundation of China (82072798, 82073873, 81803616); Jiangsu province key research and development program: Social development project (BE2021653); Natural Science Foundation of Jiangsu Province, China (BK20201172), Key project of Jiangsu Health Commission (ZDB2020016); Research and Practice Innovation Program for Postgraduates in Jiangsu (SJCX19_0183); School-level scientific research project of Jiangsu Health Vocational College (JKC202022).

## Conflict of Interest

The authors declare that the research was conducted in the absence of any commercial or financial relationships that could be construed as a potential conflict of interest.

## Publisher’s Note

All claims expressed in this article are solely those of the authors and do not necessarily represent those of their affiliated organizations, or those of the publisher, the editors and the reviewers. Any product that may be evaluated in this article, or claim that may be made by its manufacturer, is not guaranteed or endorsed by the publisher.

## Supplementary Material

Supplementary Figure 1
**(A, B)** The expression of hsa_circ_0001367 in T98G and LN229 cells transfected with vector, circ_0001367, si-NC, si-circ_0001367-1 or circ_0001367-2. **(C)** The expression of hsa_circ_0001367 in T98G and LN229 cells transfected with vector, circ_0001367, circ_0001367+miR-NC or circ_0001367+miR-545-3p. **(D)** LUZP1 expression in T98G and LN229 cells transfected with anti-NC, anti-mir-545-3p, anti-miR-545-3p+si-NC or anti-miR-545-3p+si-LUZP1.

## References

[1] Huang H Zhang W Fang Y Hong J Su S Lai X. Overall Survival Prediction for Gliomas Using a Novel Compound Approach. Front Oncol (2021) 11:724191. doi: 10.3389/fonc.2021.724191 34490121 PMC8416476

[2] Huang Q Lian C Dong Y Zeng H Liu B Xu N. SNAP25 Inhibits Glioma Progression by Regulating Synapse Plasticity *via* GLS-Mediated Glutaminolysis. Front Oncol (2021) 11:698835. doi: 10.3389/fonc.2021.698835 34490096 PMC8416623

[3] Chang YZ Li GZ Pang B Zhang KN Zhang XH Wang YZ. Transcriptional Characteristics of IDH-Wild Type Glioma Subgroups Highlight the Biological Processes Underlying Heterogeneity of IDH-Wild Type WHO Grade IV Gliomas. Front Cell Dev Biol (2020) 8:580464. doi: 10.3389/fcell.2020.580464 33195221 PMC7642517

[4] Xiao ZZ Wang ZF Lan T Huang WH Zhao YH Ma C. Carmustine as a Supplementary Therapeutic Option for Glioblastoma: A Systematic Review and Meta-Analysis. Front Neurol (2020) 11:1036. doi: 10.3389/fneur.2020.01036 33041980 PMC7527463

[5] Ji X Ding F Gao J Huang X Liu W Wang Y. Molecular and Clinical Characterization of a Novel Prognostic and Immunologic Biomarker FAM111A in Diffuse Lower-Grade Glioma. Front Oncol (2020) 10:573800. doi: 10.3389/fonc.2020.573800 33194678 PMC7649369

[6] Li X Wang J Long H Lin W Wang H Chen Y. circCDYL2, Overexpressed in Highly Migratory Colorectal Cancer Cells, Promotes Migration by Binding to Ezrin. Front Oncol (2021) 11:716073. doi: 10.3389/fonc.2021.716073 34485151 PMC8416158

[7] Mo Y Wang Y Zhang S Xiong F Yan Q Jiang X. Circular RNA Circrnf13 Inhibits Proliferation and Metastasis of Nasopharyngeal Carcinoma *via* SUMO2. Mol Cancer (2021) 20(1):112. doi: 10.1186/s12943-021-01409-4 34465340 PMC8406723

[8] Belter A Popenda M Sajek M Wozniak T Naskret-Barciszewska MZ Szachniuk M. A New Molecular Mechanism of RNA Circularization and the microRNA Sponge Formation. J Biomol Struct Dyn (2020) 1–8. doi: 10.1080/07391102.2020.1844802 33200684

[9] Rajappa A Banerjee S Sharma V Khandelia P. Circular RNAs: Emerging Role in Cancer Diagnostics and Therapeutics. Front Mol Biosci (2020) 7:577938. doi: 10.3389/fmolb.2020.577938 33195421 PMC7655967

[10] Taulli R Loretelli C Pandolfi PP. From Pseudo-ceRNAs to circ-ceRNAs: A Tale of Cross-Talk and Competition. Nat Struct Mol Biol (2013) 20(5):541–3. doi: 10.1038/nsmb.2580 PMC4141855 23649362

[11] Guarnerio J Zhang Y Cheloni G Panella R Mae Katon J Simpson M. Intragenic Antagonistic Roles of Protein and circRNA in Tumorigenesis. Cell Res (2019) 29(8):628–40. doi: 10.1038/s41422-019-0192-1 PMC6796857 31209250

[12] Guarnerio J Bezzi M Jeong JC Paffenholz SV Berry K Naldini MM. Oncogenic Role of Fusion-circRNAs Derived From Cancer-Associated Chromosomal Translocations. Cell (2016) 166(4):1055–6. doi: 10.1016/j.cell.2016.07.035 27518567

[13] Liu Z Guo S Sun H Bai Y Song Z Liu X. Circular RNA CircHIPK3 Elevates CCND2 Expression and Promotes Cell Proliferation and Invasion Through miR-124 in Glioma. Front Genet (2020) 11:1013. doi: 10.3389/fgene.2020.01013 33005182 PMC7485042

[14] Lou J Hao Y Lin K Lyu Y Chen M Wang H. Circular RNA CDR1as Disrupts the P53/MDM2 Complex to Inhibit Gliomagenesis. Mol Cancer (2020) 19(1):138. doi: 10.1186/s12943-020-01253-y 32894144 PMC7487905

[15] Yang Y Gao X Zhang M Yan S Sun C Xiao F. Novel Role of FBXW7 Circular RNA in Repressing Glioma Tumorigenesis. J Natl Cancer Inst (2018) 110(3):301–15. doi: 10.1093/jnci/djx166 PMC6019044 28903484

[16] Ghafouri-Fard S Gholipour M Taheri M Shirvani Farsani Z. MicroRNA Profile in the Squamous Cell Carcinoma: Prognostic and Diagnostic Roles. Heliyon (2020) 6(11):e05436. doi: 10.1016/j.heliyon.2020.e05436 33204886 PMC7653070

[17] Pisarska J Baldy-Chudzik K. MicroRNA-Based Fingerprinting of Cervical Lesions and Cancer. J Clin Med (2020) 9(11):3668. doi: 10.3390/jcm9113668 PMC7698009 33203149

[18] Ludwig S Sharma P Wise P Sposto R Hollingshead D Lamb J. mRNA and miRNA Profiles of Exosomes From Cultured Tumor Cells Reveal Biomarkers Specific for HPV16-Positive and HPV16-Negative Head and Neck Cancer. Int J Mol Sci (2020) 21(22):8570. doi: 10.3390/ijms21228570 PMC7698015 33202950

[19] Duica F Condrat CE Danila CA Boboc AE Radu MR Xiao J. MiRNAs: A Powerful Tool in Deciphering Gynecological Malignancies. Front Oncol (2020) 10:591181. doi: 10.3389/fonc.2020.591181 33194751 PMC7646292

[20] Sun G Li Z He Z Wang W Wang S Zhang X. Circular RNA MCTP2 Inhibits Cisplatin Resistance in Gastric Cancer by miR-99a-5p-Mediated Induction of MTMR3 Expression. J Exp Clin Cancer Res (2020) 39(1):246. doi: 10.1186/s13046-020-01758-w 33198772 PMC7670601

[21] Bozal-Basterra L Gonzalez-Santamarta M Muratore V Bermejo-Arteagabeitia A Da Fonseca C Barroso-Gomila O. LUZP1, A Novel Regulator of Primary Cilia and the Actin Cytoskeleton, Is a Contributing Factor in Townes-Brocks Syndrome. Elife (2020) 9:e55957. doi: 10.7554/eLife.55957 PMC7363444 32553112

[22] Poel D Boyd LNC Beekhof R Schelfhorst T Pham TV Piersma SR. Proteomic Analysis of miR-195 and miR-497 Replacement Reveals Potential Candidates That Increase Sensitivity to Oxaliplatin in MSI/P53wt Colorectal Cancer Cells. Cells (2019) 8(9):1111. doi: 10.3390/cells8091111 PMC6770888 31546954

[23] Hsu CY Chang NC Lee MW Lee KH Sun DS Lai C. LUZP Deficiency Affects Neural Tube Closure During Brain Development. Biochem Biophys Res Commun (2008) 376(3):466–71. doi: 10.1016/j.bbrc.2008.08.170 18801334

[24] Long Z Gong F Li Y Fan Z Li J. Circ_0000285 Regulates Proliferation, Migration, Invasion and Apoptosis of Osteosarcoma by miR-409-3p/IGFBP3 Axis. Cancer Cell Int (2020) 20:481. doi: 10.1186/s12935-020-01557-5 33041662 PMC7539413

[25] Li D Patel CB Xu G Iagaru A Zhu Z Zhang L. Visualization of Diagnostic and Therapeutic Targets in Glioma With Molecular Imaging. Front Immunol (2020) 11:592389. doi: 10.3389/fimmu.2020.592389 33193439 PMC7662122

[26] Bejugam PR Das A Panda AC. Seeing Is Believing: Visualizing Circular RNAs. Noncoding RNA (2020) 6(4):45. doi: 10.3390/ncrna6040045 PMC7712394 33187156

[27] Liu L Zhang P Dong X Li H Li S Cheng S. Circ_0001367 Inhibits Glioma Proliferation, Migration and Invasion by Sponging miR-431 and Thus Regulating NRXN3. Cell Death Dis (2021) 12(6):536. doi: 10.1038/s41419-021-03834-1 34035217 PMC8149867

[28] Fan D Wang C Wang D Zhang N Yi T. Circular RNA Circ_0000039 Enhances Gastric Cancer Progression Through miR-1292-5p/DEK Axis. Cancer Biomark (2021) 30(2):167–77. doi: 10.3233/CBM-201754 PMC12499984 33104023

[29] Li H He L Tuo Y Huang Y Qian B. Circular RNA hsa_circ_0000282 Contributes to Osteosarcoma Cell Proliferation by Regulating miR-192/XIAP Axis. BMC Cancer (2020) 20(1):1026. doi: 10.1186/s12885-020-07515-8 33097010 PMC7583201

[30] Liang Y Shi J He Q Sun G Gao L Ye J. Hsa_circ_0026416 Promotes Proliferation and Migration in Colorectal Cancer *via* miR-346/NFIB Axis. Cancer Cell Int (2020) 20:494. doi: 10.1186/s12935-020-01593-1 33061846 PMC7549246

[31] Li Z Zheng J Lin W Weng J Hong W Zou J. Circular RNA hsa_Circ_0001785 Inhibits the Proliferation, Migration and Invasion of Breast Cancer Cells *In Vitro* and *In Vivo* by Sponging miR-942 to Upregulate SOCS3. Cell Cycle (2020) 19(21)2811–25. doi: 10.1080/15384101.2020.1824717 PMC7714452 33054543

[32] Li S Pei Y Wang W Liu F Zheng K Zhang X. Circular RNA 0001785 Regulates the Pathogenesis of Osteosarcoma as a ceRNA by Sponging miR-1200 to Upregulate HOXB2. Cell Cycle (2019) 18(11):1281–91. doi: 10.1080/15384101.2019.1618127 PMC6592237 31116090

[33] Zhang W Zhang S. Downregulation of circRNA_0000285 Suppresses Cervical Cancer Development by Regulating Mir197-3p-ELK1 Axis. Cancer Manag Res (2020) 12:8663–74. doi: 10.2147/CMAR.S253174 PMC7509321 32982457

[34] Yang D Jin Y Cheng S Yang Y. The Interaction Between Circular RNA hsa_circ_0000285 and miR-599 in Thyroid Cancer. Eur Rev Med Pharmacol Sci (2020) 24(13):7219. doi: 10.26355/eurrev_202007_21870 32706057

[35] Shi J Xu X Zhang D Zhang J Yang H Li C. Long Non-Coding RNA PTPRG-AS1 Promotes Cell Tumorigenicity in Epithelial Ovarian Cancer by Decoying microRNA-545-3p and Consequently Enhancing HDAC4 Expression. J Ovarian Res (2020) 13(1):127. doi: 10.1186/s13048-020-00723-7 33099316 PMC7585679

[36] Hao R Wang B Wang H Huo Y Lu Y. lncRNA TUG1 Promotes Proliferation and Differentiation of Osteoblasts by Regulating the miR-545-3p/CNR2 Axis. Braz J Med Biol Res (2020) 53(11):e9798. doi: 10.1590/1414-431X20209798 33053117 PMC7552904

[37] Zhong Y Wang Y Dang H Wu X. LncRNA AFAP1-AS1 Contributes to the Progression of Endometrial Carcinoma by Regulating miR-545-3p/VEGFA Pathway. Mol Cell Probes (2020) 53:101606. doi: 10.1016/j.mcp.2020.101606 32504788

[38] Chen S Lu S Yao Y Chen J Yang G Tu L. Downregulation of hsa_circ_0007580 Inhibits Non-Small Cell Lung Cancer Tumorigenesis by Reducing miR-545-3p Sponging. Aging (Albany NY) (2020) 12(14):14329–40. doi: 10.18632/aging.103472 PMC7425484 32681720

[39] Li H Liu F Qin W. Circ_0072083 Interference Enhances Growth-Inhibiting Effects of Cisplatin in Non-Small-Cell Lung Cancer Cells *via* miR-545-3p/CBLL1 Axis. Cancer Cell Int (2020) 20:78. doi: 10.1186/s12935-020-1162-x 32190002 PMC7066755

