# Supplementary figures and images for: The Circ_0001367/miR-545-3p/LUZP1 Axis Regulates Cell Proliferation, Migration and Invasion in Glioma Cells
Source: Front Oncol. 2021 Nov 18;11:781471. doi: 10.3389/fonc.2021.781471 (PMC8637337; doi:10.3389/fonc.2021.781471)

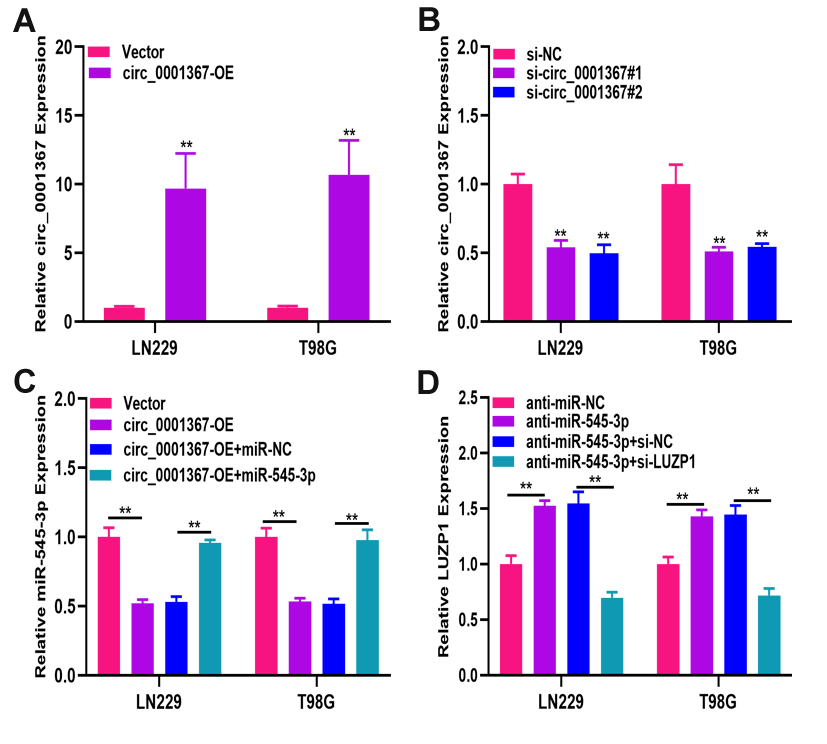

Supplement: Supplementary Figure 1 — (A, B) The expression of hsa_circ_0001367 in T98G and LN229 cells transfected with vector, circ_0001367, si-NC, si-circ_0001367-1 or circ_0001367-2. (C) The expression of hsa_circ_0001367 in T98G and LN229 cells transfected with vector, circ_0001367, circ_0001367+miR-NC or circ_0001367+miR-545-3p. (D) LUZP1 expression in T98G and LN229 cells transfected with anti-NC, anti-mir-545-3p, anti-miR-545-3p+si-NC or anti-miR-545-3p+si-LUZP1. [file Image_1.tif]

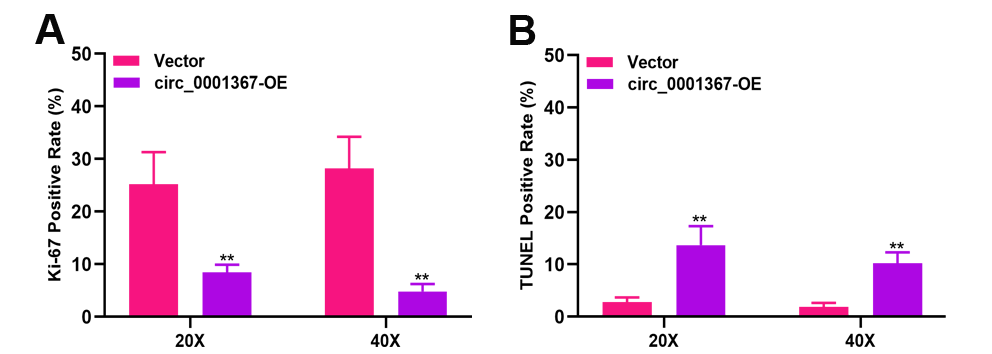

Supplement: Supplementary file 2 [file Image_2.tif]
